# Supplementary material for: Clustering and Recognition of Spatiotemporal Features Through Interpretable Embedding of Sequence to Sequence Recurrent Neural Networks
Source: Front Artif Intell. 2020 Sep 30;3:70. doi: 10.3389/frai.2020.00070 (PMC7861310; doi:10.3389/frai.2020.00070)
Supplement: Supplementary file 1 [file Data_Sheet_1.PDF]

Clustering and Recognition of Spatiotemporal Features through Interpretable Embedding of Sequence to Sequence Recurrent Neural Networks
Supplementary Material

Kun Su[1], Eli Shlizerman [1,2]
[1]Department of Electrical & Computer Engineering
[2]Department of Applied Mathematics
University of Washington
Seattle, WA 98195
suk4,shlizee@uw.edu

POD vs t-SNE

While TSNE embedding has been shown useful in several domains, in particular in the text domain, it is not fully compatible with real time series data. We show two examples in Fig.1 (sitting movement) and Fig.2 (walking movement) comparing the POD embedding that we use, with the t-SNE embedding. In both cases, it can be observed that our method represents the sequences, their pointwise continuity and evolution in a more intuitive and interpretable way than t-SNE. For example, for the sitting movement, the POD embedding accurately shows denser points (faster movement) in the beginning and the end of the movement as expected.

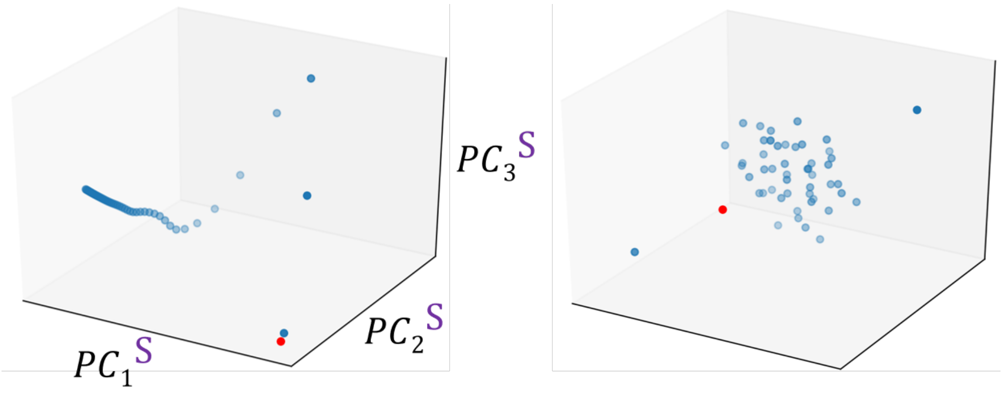

Figure 1: POD (left) vs t-SNE (right) embedding of **sitting** example: The red point indicates the first state of decoder.

More experimental results on Human 3.6M

We observed that the encoder length is a sensitive parameter in terms of clustering accuracy. In Table 1 we examine how the clustering accuracy depends on the length of the encoder with fixed decoder length (50).

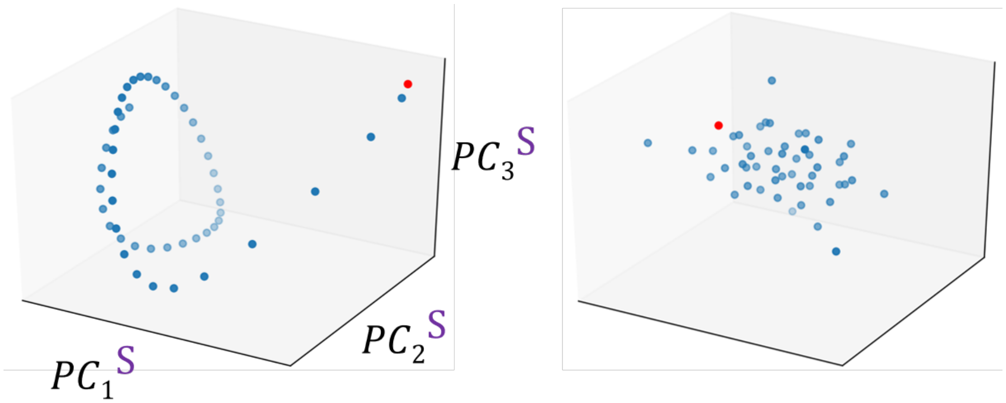

Figure 2: POD (left) vs t-SNE (right) embedding of **walking** example: The red point indicates the first state of decoder.

Table 1: Clustering dependence on encoder length

Encoder Length	Dimension	ARI (%)	
		Mean	Std
25	dim = 3	73.9	6.8
	dim = 10	87.6	4.9
	dim = 1024	96.7	4.2
50	dim = 3	90.8	6.6
	dim = 10	96.3	3.7
	dim = 1024	99.1	2.4
75	dim = 3	79.5	6.0
	dim = 10	90.7	4.5
	dim = 1024	97.3	3.5
100	dim = 3	85.1	2.2
	dim = 10	83.7	2.6
	dim = 1024	86.5	5.6

Our experiments show that, on one hand, the encoder length should be long enough to capture necessary information to fully encode the action sequence. On the other hand, extremely long encoder lengths may hamper the clustering performance. These are attributed to inherent instabilities in sequence neural networks such as the vanishing gradient problem.

Unsupervised action recognition

We evaluate the clustering property of RNN-Seq2Seq on CMU Motion capture dataset. We provide two videos (cf. E1) and figures (cf. E2) to demonstrate our results [1].

E1: Concatenated Sequences

We provide three video examples in zip file for demonstration. Each video includes the action in x, y, z space on the top left, the decoder states representation in 3D on the top right, predicted labels and ground truth labels on the bottom. Each color indicates one distinct action. **Video_1** shows the simplest case that

[1] The code with these evaluations is available at https://shlizee.github.io/interpretseq2seq/

includes single walking, running and jumping motion. **Video_2** demonstrates the case of repeatable walking, running, and jumping motion. **Video_3** shows the case of walking, running, jumping, soccer and basketball motion.

E2: Subject number 86

Clustering results of CMU subject 86 are shown in Figs. 3, 4. Each color represents one distinct action. The labels (Predict) and (GT) indicate our prediction compared with the ground truth respectively. Along with comparing the time-segmentation of actions using colors we also show the total frame-level accuracy in percentage to the right of each comparison (Acc). For 10 out of 14 tests we obtain 100% accuracy and 94.75% overall accuracy across all tests. We find that failure cases are mainly credited to highly similar movements and have similar continuity profiles. In tests 4, 5 and 6, the action "punch" has been clustered with other continuous hand-dominant motions ("drink", "cheer", "slap"). In test 8, the model fails to separate "leg kick back" from "run" which are both continuous leg-dominant motions.

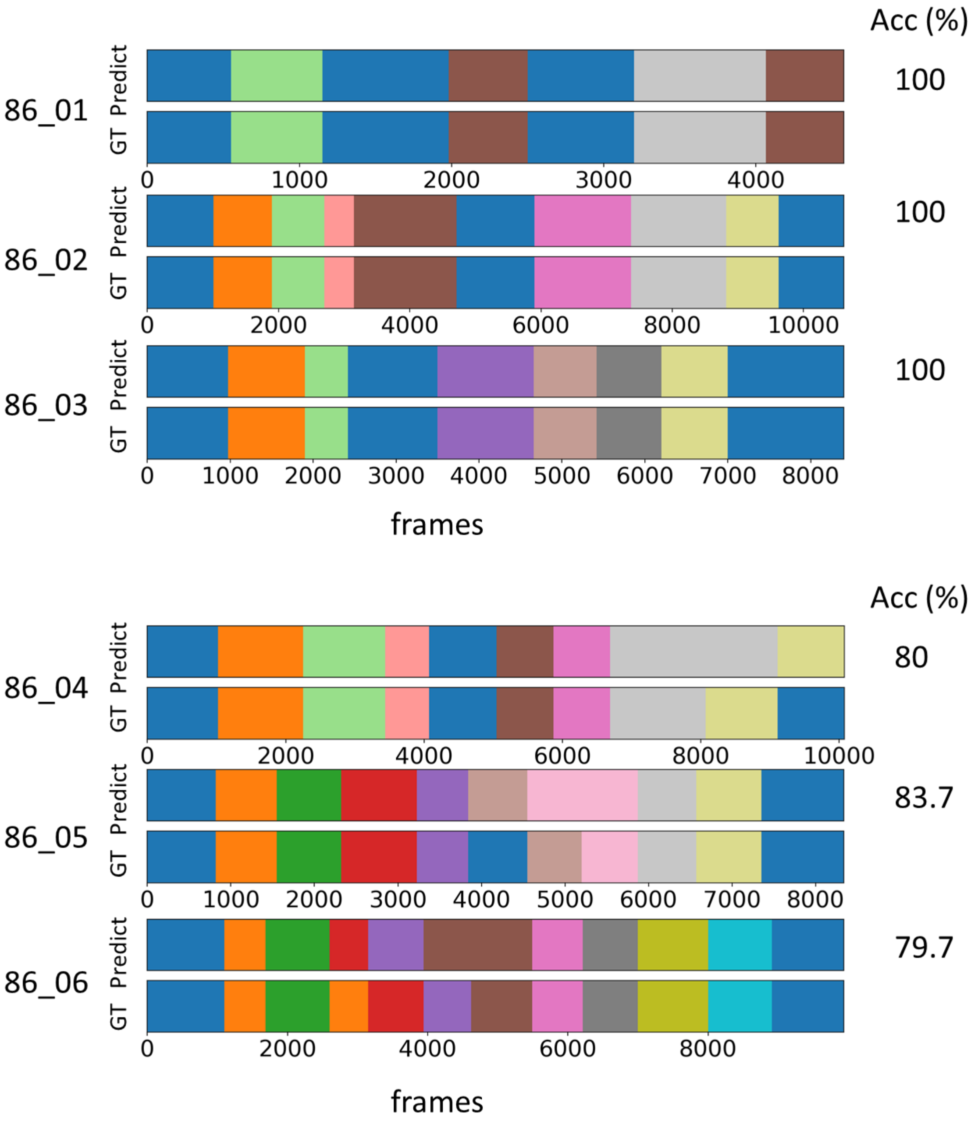

Figure 3: Results of tests 1 to 6

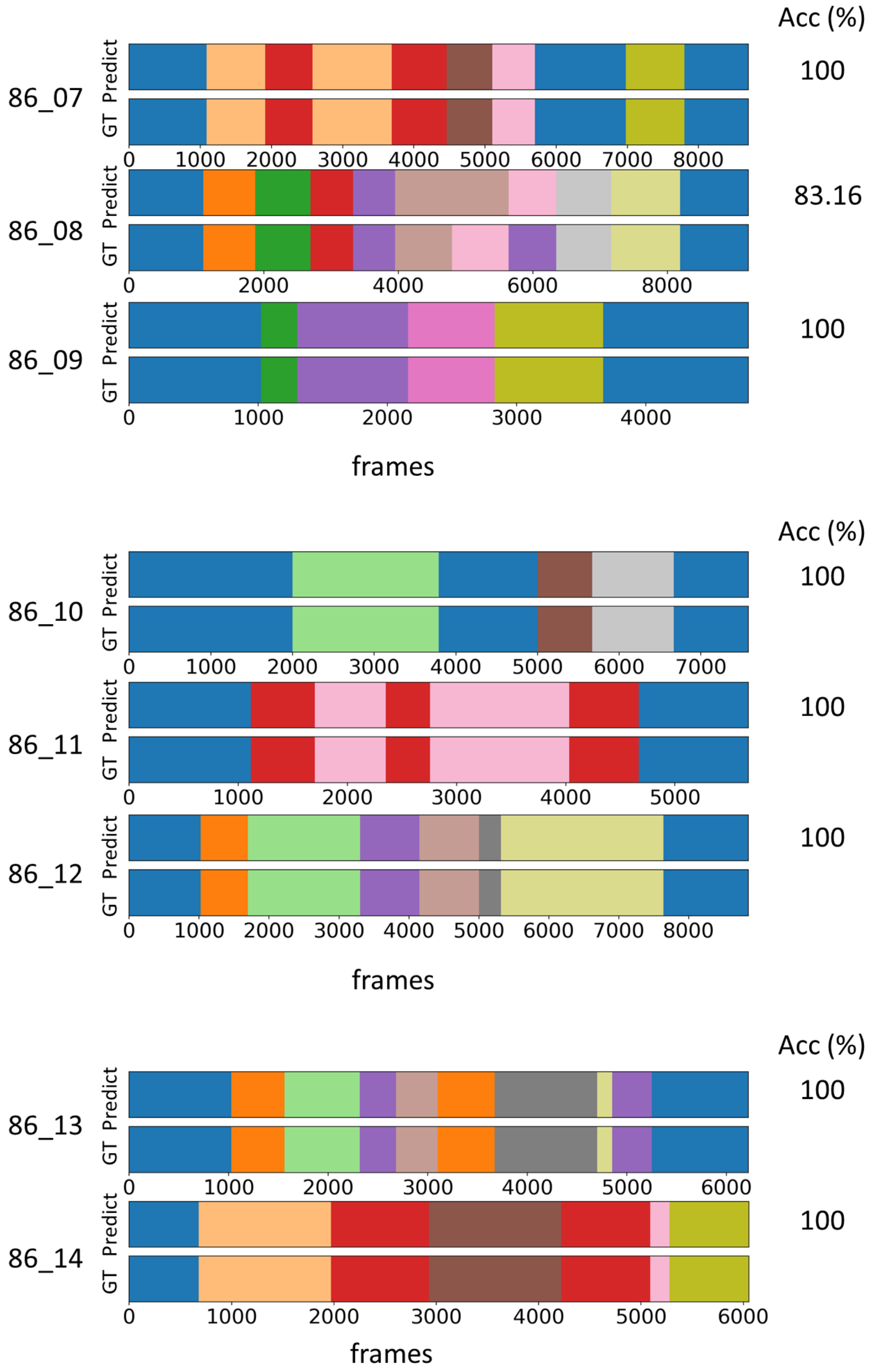

Figure 4: Results of tests 7 to 14
